# Rhombencephalitis: pictorial essay*

**DOI:** 10.1590/0100-3984.2015.0189

**Published:** 2016

**Authors:** Líllian Gonçalves Campos, Régis Augusto Reis Trindade, Ângela Faistauer, Juliano Adams Pérez, Leonardo Modesti Vedolin, Juliana Ávila Duarte

**Affiliations:** 1 MD, Neuroradiologist at Hospital Moinhos de Vento and the Hospital de Clínicas de Porto Alegre (HCPA), Porto Alegre, RS, Brazil.; 2 MD, Resident in Radiology and Diagnostic Imaging at the Hospital de Clínicas de Porto Alegre (HCPA), Porto Alegre, RS, Brazil.; 3 MD, Radiologist at Hospital Escola da Universidade Federal de Pelotas (UFPel), Pelotas, RS, Brazil.; 4 PhD, MD, Neuroradiologist at the Hospital de Clínicas de Porto Alegre (HCPA), Porto Alegre, RS, Brazil.

**Keywords:** Encephalitis, Rhombencephalon, Magnetic resonance imaging

## Abstract

The term rhombencephalitis refers to inflammatory diseases affecting the hindbrain (brainstem and cerebellum). Rhombencephalitis has a wide variety of etiologies, including infections, autoimmune diseases, and paraneoplastic syndromes. Infection with bacteria of the genus *Listeria* is the most common cause of rhombencephalitis. Primary rhombencephalitis caused by infection with *Listeria* spp. occurs in healthy young adults. It usually has a biphasic time course with a flu-like syndrome, followed by brainstem dysfunction; 75% of patients have cerebrospinal fluid pleocytosis, and nearly 100% have an abnormal brain magnetic resonance imaging scan. However, other possible causes of rhombencephalitis must be borne in mind. In addition to the clinical aspects, the patterns seen in magnetic resonance imaging can be helpful in defining the possible cause. Some of the reported causes of rhombencephalitis are potentially severe and life threatening; therefore, an accurate initial diagnostic approach is important to establishing a proper early treatment regimen. This pictorial essay reviews the various causes of rhombencephalitis and the corresponding magnetic resonance imaging findings, by describing illustrative confirmed cases.

## Abstract (pt)

O termo rombencefalite se refere a doenças inflamatórias que afetam o rombencéfalo (tronco cerebral e cerebelo). Rombencefalites possuem grande variedade de causas, incluindo infecciosas, autoimunes e síndromes paraneoplásicas. *Listeria* é a causa mais comum das rombencefalites infecciosas. A rombencefalite primária por *Listeria* ocorre em adultos jovens e saudáveis, com um curso de tempo bifásico, como uma síndrome gripal acompanhada de disfunção do tronco cerebral. Em 75% dos pacientes manifesta-se pleiocitose no líquido cefalorraquidiano e em quase 100% a ressonância magnética cerebral é anormal. Mas há outras causas possíveis para rombencefalites que devem ser lembradas. Além de aspectos clínicos, os padrões de imagem encontrados na ressonância magnética podem ser úteis na definição da possível causa. Algumas das causas descritas de rombencefalites são potencialmente graves e fatais; assim, uma abordagem diagnóstica inicial precisa é importante para estabelecer um tratamento precoce adequado. Este ensaio iconográfico revisa as diversas causas de rombencefalites e os seus achados de ressonância magnética, por meio de casos ilustrativos confirmados.

## INTRODUCTION

The term rhombencephalitis refers to inflammatory diseases affecting the hindbrain (brainstem and cerebellum). Rhombencephalitis has a great variety of etiologies, some of them serious and potentially fatal without early, appropriate treatment. The hindbrain is composed of the pons, cerebellum and medulla. In addition, the terms "brainstem" encephalitis and rhombencephalitis have often been used interchangeably^(1,2)^.

In this pictorial essay, we reviewed the medical records of patients admitted to the Hospital de Clínicas de Porto Alegre, in the city of Porto Alegre, RS, Brazil, between November 2009 and November 2013.

## CAUSES AND CLINICAL FINDINGS

The causes of rhombencephalitis can be divided into infectious diseases, autoimmune diseases, and paraneoplastic syndromes. The most common infectious causes are bacteria of the genus *Listeria*, enterovirus 71, and the herpes viruses^(1)^. The most common autoimmune cause is Behcet's disease. There have been isolated reports of cases of rhombencephalitis caused by systemic lupus erythematosus and relapsing polychondritis^(3)^.

Cases of rhombencephalitis caused by paraneoplastic syndromes have been associated with anti-Yo, anti-Tr, anti-Hu, anti-Ri, anti-Ma, and anti-amphiphysin antibodies. In most such cases, the underlying cause was small cell lung cancer^(1,2,4)^.

*Listeria monocytogenes* is a major bacterial pathogen, especially in newborns, immunocompromised patients, the elderly, pregnant women, and, less frequently, previously healthy individuals. The incubation period for noninvasive *Listeria*-associated gastroenteritis is substantially shorter than is that for the invasive form of the disease^(4)^.

Behcet's disease is a multisystem form of idiopathic vasculitis. The classic triad of oral ulcers, genital ulcers, and uveitis was originally described by Behcet in 1937^(3)^.

Central nervous system (CNS) necrosis secondary to radiation is a severe, uncommon adverse reaction to radiotherapy, typically occurring one to three years after the end of treatment ^(5)^.

Cerebral aspergillosis is a rare condition that primarily affects immunocompromised hosts. Its prevalence has increased with the use of intensive chemotherapy regimens, corticosteroid therapy, and transplants^(6)^.

Paracoccidioidomycosis is a systemic fungal infection, endemic to Central and South America, which affects the CNS in nearly 10% of cases. The neurological involvement in paracoccidioidomycosis includes a granulomatous form and a meningeal, or pseudotumor, form^(1,7)^.

In patients with systemic lupus erythematosus, CNS symptoms are often observed during the course of the disease, the reported prevalence of such manifestations ranging from 17% to 75%. MRI is considered the gold standard for the evaluation of these manifestations in clinical practice^(1,2)^.

## IMAGING FINDINGS

In previous studies, MRI scans were abnormal in 100% of cases of *Listeria*-associated rhombencephalitis (100% infratentorial, 50% supratentorial)^(1,2)^. Among cases of viral rhombencephalitis, MRI scans are abnormal in 70-75% of the cases caused by enterovirus 71, in 67% of the cases caused by combined herpes (herpes simplex virus, Epstein-Barr virus, cytomegalovirus, varicella zoster virus, and human herpes virus 6), and in nearly 100% of the cases caused by Behcet's disease^(1,2,5)^.

### Listeria

**Figure 1:**
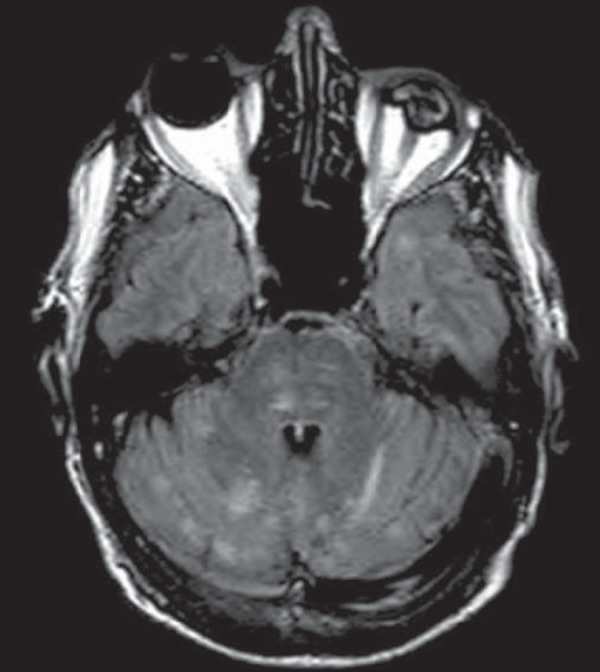
*Listeria*. Axial FLAIR MRI sequence showing irregular, ill-defined hyperintense lesions.

**Figure 2:**
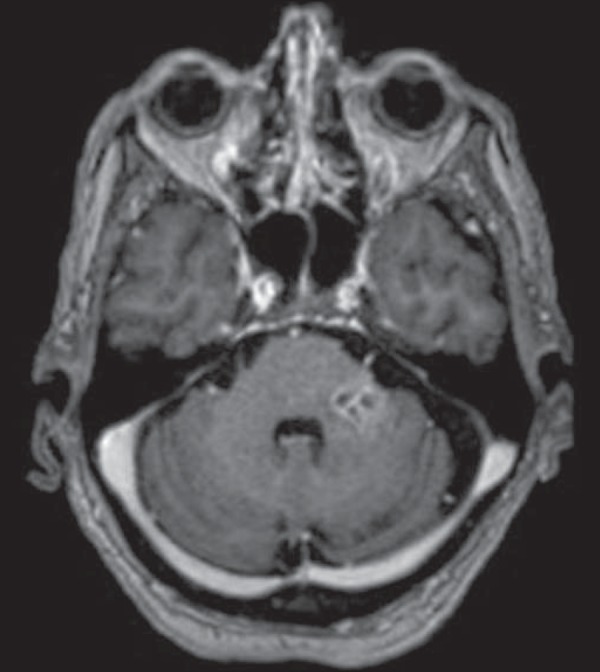
*Listeria*. Axial gadolinium contrast-enhanced T1-weighted sequence showing multiple lesions with ring enhancement in the left pons.

Most cases of *Listeria*-associated rhombencephalitis present imaging findings similar to those of other causes. On T2-weighted and fluid-attenuated inversion recovery (FLAIR) MRI sequences, areas with a hyperintense signal are seen in the brain stem, cerebellum, and upper cervical spinal cord. Unlike in rhombencephalitis of other causes, abscesses with ring enhancement, which can be useful in making a diagnosis of *Listeria*-associated rhombencephalitis^(1,4)^, can be seen in those same locations (Figures 1 and 2).

### Human herpes virus 6

**Figure 3:**
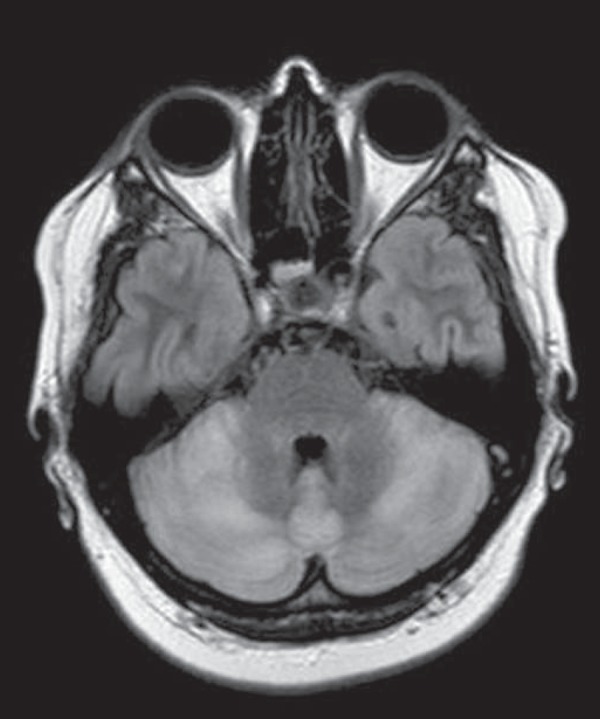
Herpes virus. FLAIR MRI sequence showing hyperintense lesions in the cerebellum, involving white and gray matter.

**Figure 4:**
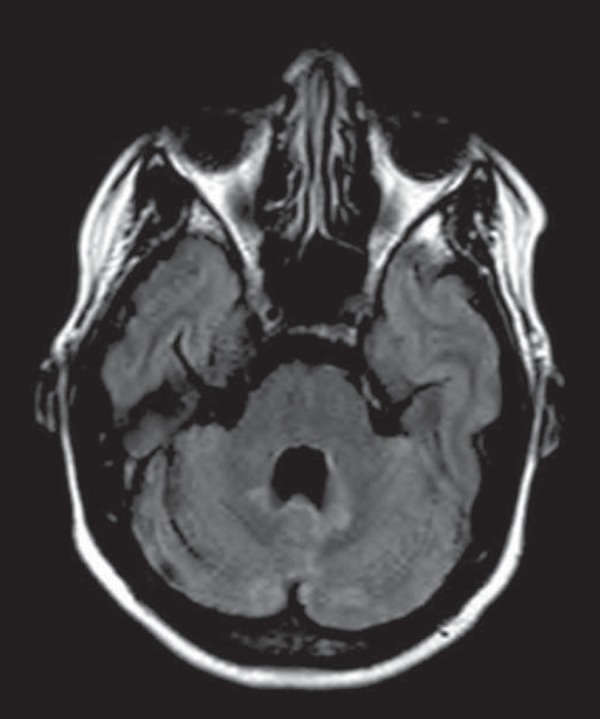
Epstein-Barr virus, cytomegalovirus, and herpes simplex virus. Axial FLAIR MRI sequences showing irregular, bilateral, asymmetric hyperintense regions that affect the pons and the cerebellar peduncles, without a significant mass effect.

In cases of rhombencephalitis caused by human herpes virus 6, MRI scans show areas with a hyperintense signal^(8)^, not only in the cerebellum but also in the thalami, putamina, and insular cortex (Figures 3 and 4).

### Neuro-Behçet's disease

**Figure 5:**
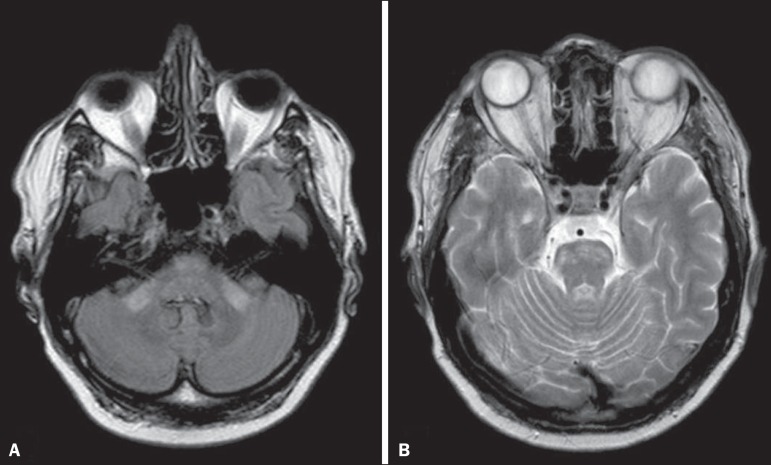
Neuro-Behçet's disease. Axial FLAIR MRI sequence (**A**) and axial T2-weighted MRI sequence (**B**) showing, at the base of the cerebral peduncle, heterogeneous lesion bilaterally at the mesodiencephalic junction with extensive swelling, sparing the red nucleus. Note the cranial extent of the perilesional edema.

**Figure 6:**
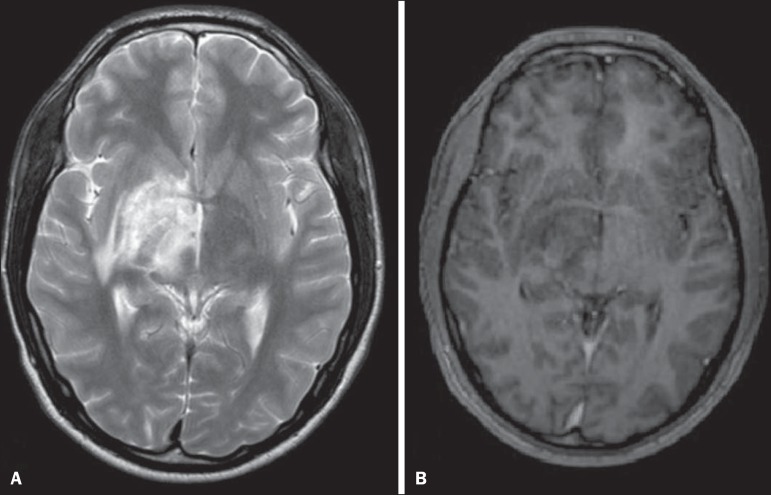
Neuro-Behçet's disease. MRI T2-weighted sequence (**A**) and T1-weighted sequence (**B**) showing a heterogeneous lesion in the mesodiencephalic junction, with extensive edema without enhancement after injection of gadolinium-based contrast medium in the right pontine-mesencephalic region and thalamus.

In neuro-Behçet's disease, MRI findings show a predilection for involvement of the brainstem-diencephalon and a tendency to resolve over time. Cerebral venous thrombosis (Figures 5 and 6) is another common neuroimaging finding in neuro-Behçet's disease^(3)^.

### Radiation necrosis

**Figure 7:**
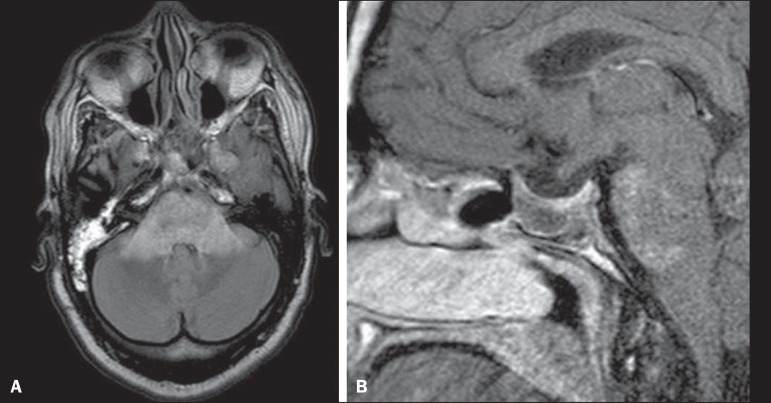
Radiation necrosis. Axial FLAIR MRI sequence (**A**) and sagittal gadolinium contrast-enhanced T1-weighted sequence (**B**) of a 36-year-old female, six months after irradiation of a pituitary macroadenoma, showing extensive edema at the mesodiencephalic junction and enhancement of the area of the radiation field.

In cases of necrosis secondary to radiotherapy, the lesions usually affect the white matter more than the gray matter and the cerebral cortex is relatively unaffected. On MRI scans, such lesions exhibit low signal intensity on T1-weighted sequences and high signal intensity on T2-weighted sequences and can extend beyond the irradiated tissue, corresponding to cerebral edema. The lesions are typically rounded or irregular, with a "Swiss cheese" or "soap bubble" appearance. Patterns of contrast enhancement vary in aspect, with small nodules with ring enhancement ^(5)^. Water diffusion sequences, although not very useful in this case, typically show facilitated diffusion, useful in distinguishing brain abscesses, which usually feature restricted diffusion, and some tumors. Because suspicious intracranial lesions are most often detected in most patients who have undergone radiotherapy of the brain, the main diagnostic objective is to differentiate between radiation necrosis and residual neoplasia or recurrence of the neoplasia (Figure 7).

### Tuberculosis

**Figure 8:**
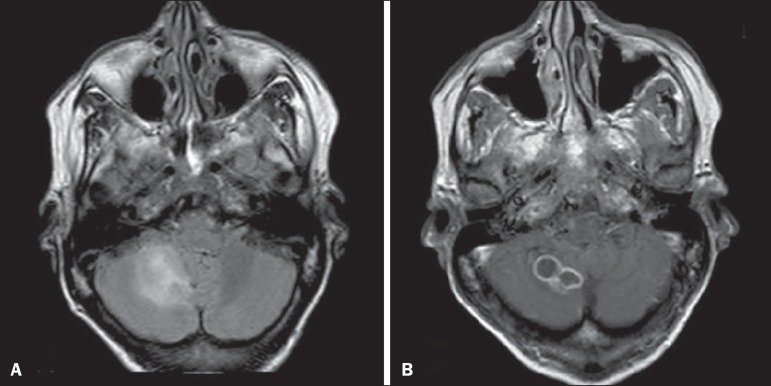
Tuberculosis. **A:** Axial FLAIR MRI sequence showing lesions with vasogenic edema. **B:** Multiple lesions with ring enhancement in the right cerebral hemisphere, with vasogenic edema and central hypointensity on gadolinium contrast-enhanced T1-weighted sequences.

**Figure 9:**
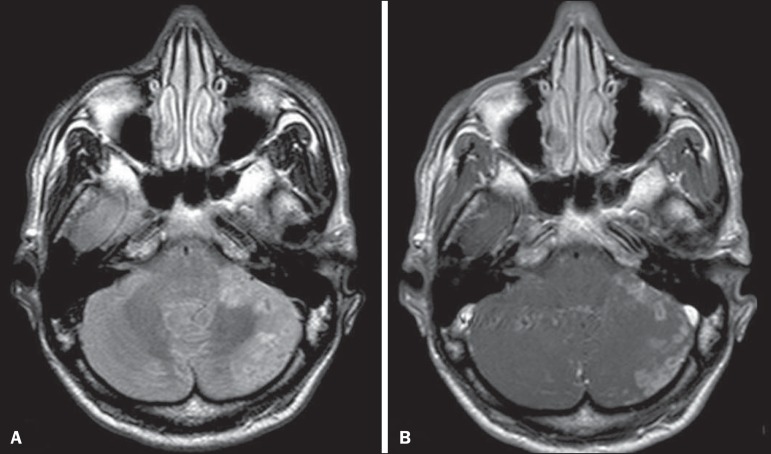
Tuberculosis. **A:** Axial FLAIR MRI sequence showing lesions with vasogenic edema. **B:** Multiple lesions with ring enhancement confluent, mainly in the left cerebral hemisphere, with leptomeningeal enhancement.

The involvement of the basal cisterns with leptomeningeal enhancement after intravenous injection of contrast is the finding most characteristic of tuberculosis-associated rhombencephalitis. In the brain stem, the most common lesion is tuberculoma. The MRI findings include a hyperintense signal on T2-weighted and FLAIR sequences in the central portion of the lesions, with a hypointense halo and peripheral edema. After intravenous injection of gadolinium-based contrast medium, the lesions typically present peripheral or nodular enhancement (Figures 8 and 9), depending on whether there is central necrosis^(9)^.

### Progressive multifocal leukoencephalopathy

**Figure 10:**
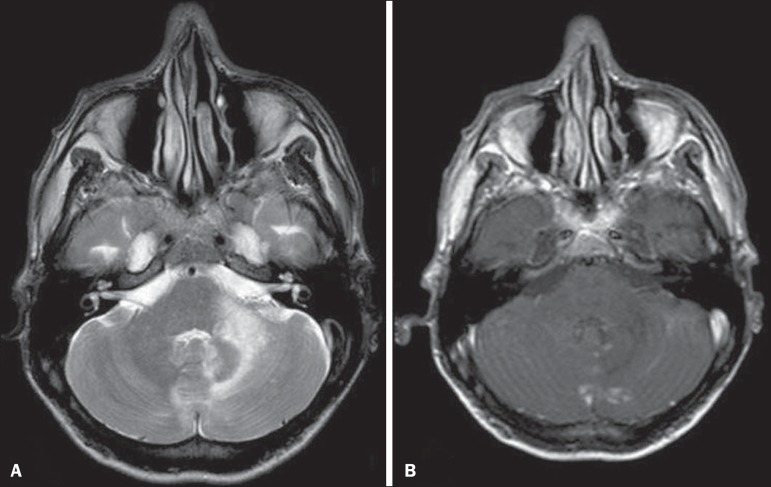
Progressive multifocal leukoencephalopathy. Axial T2-weighted MRI sequence (**A**) and axial T1-weighted MRI sequence (**B**) after injection of gadolinium-based contrast medium, showing ill-defined hyperintense lesions with irregular enhancement.

Progressive multifocal leukoencephalopathy occurs in immunocompromised patients, with symmetrical involvement of the subcortical white matter in the centrum semiovale of the parieto-occipital region, affecting the U-shaped fibers^(10)^, perilesional edema, and restricted water diffusion within the areas of active demyelination at the periphery of the lesion, and characteristically do not present enhancement after intravenous injection of gadolinium-based contrast (Figure 10).

### Aspergillosis

**Figure 11:**
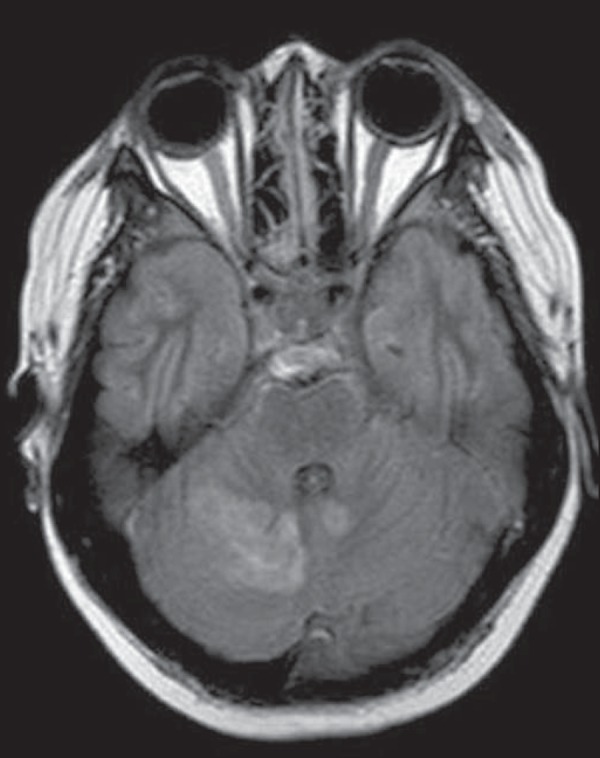
Aspergillosis. Axial FLAIR MRI sequence showing multiple lesions with intermediate signal intensity in both cerebral hemispheres, mostly on the right, with restricted diffusion of water molecules but without enhancement after injection of gadolinium-based contrast medium.

On MRI scans, the classical description of aspergillosis is of an expansile lesion with irregular contours, showing a hypointense signal on T1-weighted sequences, with homogeneous or ring enhancement after injection of gadoliniumbased contrast. In addition, a markedly hypointense signal is often seen on T2-weighted sequences. Some aspergillosis lesions present an isointense signal on the various MRI pulse sequences and have been described as being secondary to coagulation necrosis of cerebral tissue due to the vascular invasion by the fungi. Areas of signal hypointensity on T2* sequences within the walls of *Aspergillus*-induced brain lesions have also been attributed to the dense population of hyphae and to the presence of hemorrhage in the capsule, although none of the findings are specific for intracranial *Aspergillus* infections^(6)^. Another explanation for the lowintensity signal on T2* sequences is the presence of iron, manganese, and magnesium in fungal concretions (Figure 11).

### Paracoccidioidomycosis

**Figure 12:**
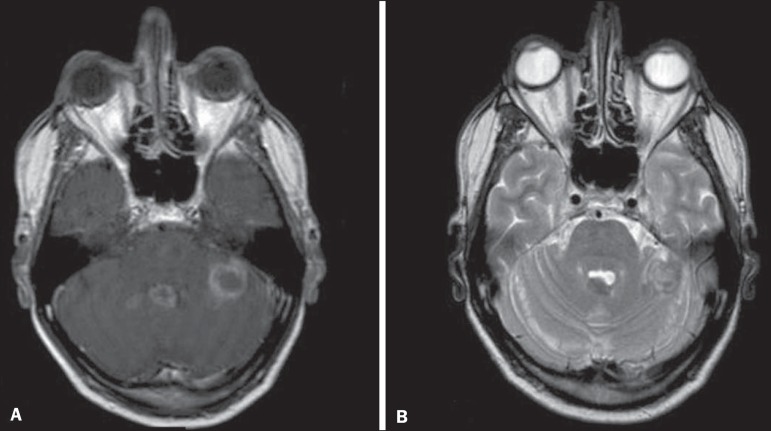
Paracoccidioidomycosis. Gadolinium contrast-enhanced T1-weighted MRI sequence (**A**) and axial T2-weighted MRI sequence (**B**) showing multiple, hypointense lesions in the posterior fossa in the T2-weighted sequences and ring enhancement in the gadolinium contrast-enhanced sequences.

The most common presentation of paracoccidioidomycosis is the presence of rounded or multiloculated lesions, predominantly hypointense on T2-weighted sequences and ring or nodular enhancement on gadolinium contrast-enhanced T1-weighted sequences. The lesions are distributed diffusely, with discrete predominance in supratentorial compartment, although infratentorial lesions have been observed, especially in the cerebellum^(7,11)^. MRI is a sensitive method for documenting paracoccidioidomycosis in the CNS, more often with multiple, supratentorial or infratentorial, rounded or lobulated lesions, on T2-weighted sequences (Figure 12).

### Systemic lupus erythematosus

**Figure 13:**
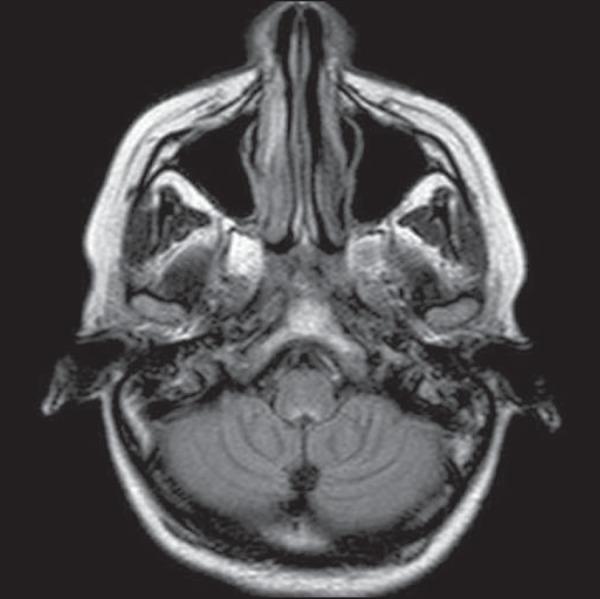
Systemic lupus erythematosus. FLAIR MRI sequence showing ill-defined, hyperintense lesions at the bulbo-medullary junction, without a mass effect and without enhancement.

The MRI findings in systemic lupus erythematosus are diverse, and signs of atrophy and signal hyperintensity in the white matter often correlate poorly with the clinical manifestations, occurring also in patients without signs or symptoms characteristic of CNS involvement^(1)^, as depicted in Figure 13.

## References

[1] Jubelt B, Mihai C, Li T (2011). Rhombencephalitis / brainstem encephalitis. Curr Neurol Neurosci Rep.

[2] Moragas M, Martínez-Yélamos S, Majós C (2011). Rhombencephalitis: a series of 97 patients. Medicine (Baltimore).

[3] Koçer N, Islak C, Siva A (1999). CNS involvement in neuro-Behçet syndrome: an MR
study. AJNR Am J Neuroradiol.

[4] Armstrong RW, Fung PC (1993). Brainstem encephalitis (rhombencephalitis) due to Listeria
monocytogenes: case report and review. Clin Infect Dis.

[5] DeSalvo MN (2012). Radiation necrosis of the pons after radiotherapy for
nasopharyngeal carcinoma: diagnosis and treatment. J Radiol Case Rep.

[6] Miaux Y, Ribaud P, Williams M (1995). MR of cerebral aspergillosis in patients who have had bone marrow
transplantation. AJNR Am J Neuroradiol.

[7] Guzmán-De-Villoria JA, Ferreiro-Argüelles C, Fernández-García P (2010). Differential diagnosis of T2 hyperintense brainstem lesions: Part
2. Diffuse lesions. Semin Ultrasound CT MR.

[8] Miura S, Kurita T, Noda K (2009). Symmetrical brainstem encephalitis caused by herpes simplex
virus. J Clin Neurosci.

[9] Gass A, Filippi M, Grossman RI (2000). The contribution of MRI in the differential diagnosis of
posterior fossa damage. J Neurol Sci.

[10] Bag AK, Curé JK, Chapman PR (2010). JC virus infection of the brain. AJNR Am J Neuroradiol.

[11] Wasenko JJ, Park BJ, Jubelt B (2002). Magnetic resonance imaging of
mesenrhombencephalitis. Clin Imaging.

